# Highly Sensitive and Cost-Effective Portable Sensor for Early Gastric Carcinoma Diagnosis

**DOI:** 10.3390/s21082639

**Published:** 2021-04-09

**Authors:** Saw-Lin Oo, Shishir Venkatesh, Vaithinathan Karthikeyan, Clement Manohar Arava, Spoorthy Pathikonda, Peter K. N. Yu, Terrence C. K. Lau, Xianfeng Chen, Vellaisamy A. L. Roy

**Affiliations:** 1 State Key Laboratory for THz and Millimeter Waves and Department of Material Science and Engineering, City University of Hong Kong, Kowloon, Hong Kong, China; kosawlinoo@gmail.com (S.L.O.); shishir.venkatesh@gmail.com (S.V.); kvecers@gmail.com (V.K.); clementmanohar7@gmail.com (C.M.A.); 2 Department of Physics, City University of Hong Kong, Kowloon, Hong Kong, China; spoorthy.709@gmail.com (S.P.); peter.yu@cityu.edu.hk (P.K.N.Y.); 3 Department of Biomedical Science, City University of Hong Kong, Kowloon, Hong Kong, China; chiklau@cityu.edu.hk; 4 Institute for Bioengineering, School of Engineering, The University of Edinburgh, King’s Buildings, Mayfield Road, Edinburgh EH9 3JL, UK; 5 James Watt School of Engineering, University of Glasgow, Glasgow G12 8QQ, UK

**Keywords:** polycarbonate membrane, cancer antigen, Oligo RNA, cancer early detection, electrical current sensor

## Abstract

Facile and efficient early detection of cancer is a major challenge in healthcare. Herein we developed a novel sensor made from a polycarbonate (PC) membrane with nanopores, followed by sequence-specific Oligo RNA modification for early gastric carcinoma diagnosis. In this design, the gastric cancer antigen CA72-4 is specifically conjugated to the Oligo RNA, thereby inhibiting the electrical current through the PC membrane in a concentration-dependent manner. The device can determine the concentration of cancer antigen CA72-4 in the range from 4 to 14 U/mL, possessing a sensitivity of 7.029 µAU^−1^mLcm^−2^ with a linear regression (R^2^) of 0.965 and a lower detection limit of 4 U/mL. This device has integrated advantages including high specificity and sensitivity and being simple, portable, and cost effective, which collectively enables a giant leap for cancer screening technologies towards clinical use. This is the first report to use RNA aptamers to detect CA72-4 for gastric carcinoma diagnosis.

## 1. Introduction

Cancer is the prevailing cause of morbidity and mortality globally, with an average of 18 million new patients every year [1]. There are over 200 forms of cancers and the most common ones are breast, lung, prostate, and colon cancers [2]. Early detection and diagnosis of cancer significantly increase the probability of successful cure and quality of life for patients and therefore it is of great importance to develop cancer detection tools. In clinic, detection of cancer tumors is usually performed by collecting and analyzing biomarkers, which are molecules released by malignant tumors into the blood, urine, or body tissues [3]. Numerous protein and gene-based biomarkers have already been used for cancer detection. For example, cancer antigen CA 74-2 is a high-molecular-weight glycosylated protein produced by tumors, which is considered to be the main indicator for gastric cancer compared to carcinoembryonic antigen (CEA) and CA19-9 because of its higher sensitivity [4]. Hence, monitoring the CA72-4 level in serum can be a valuable tool for clinical diagnosis of gastric carcinoma and evaluation of the subsequent cancer therapy [5,6].

The quantification of biochemical methods is critical for diagnosis. Many capable biological and chemical cancer biomarker detection methods have been developed and used, such as electrophoresis [7], polymerase chain reaction [8], surface-enhanced Raman spectroscopy [9], surface plasmon resonance [10], and enzyme-linked immunosorbent assay [11]. Although effective, most of these methods lack sensitivity and selectivity and are time-consuming, leading to serious disadvantages for real-time clinical diagnostic usage. In comparison, an efficient electrochemical biosensor can provide a sensitive, selective, and rapid solution to interpret the content of a cancer biomarker by directly converting a biological event into an electrical signal. In the past, a range of bio-sensing concepts and related devices have been proposed [3,12]. For instance, membrane-based electrochemical sensors were constructed as simple lab-on-a-chip instruments with reduced cost and improved accuracy. In operation, antibodies and aptamers play a crucial role in detecting cancer markers, as they can specifically bind to certain antigens. The antibodies and aptamers are often conjugated to nanoporous materials during use because of the large surface area of nanomaterials and consequently the ability of immobilization of a large amount of antigens [13,14]. Most recently, paper-based biosensors have been used to fabricate small, lightweight devices at low cost [15]. Screen-printed conductive ink-based paper substrates with specific design and structures were used according to the end-users’ requirements [16]. Paper-based cancer sensors are mostly used in the dipstick test, µPAD, and lab-on-chip devices for analyzing the antigens and cancer biomarkers [17,18,19]. In the past, many studies have demonstrated the advantages of aptamers over antibodies, such as exceptional stability under different pH, temperature, and ionic conditions, low immunogenicity and toxicity, as well as deep tumor penetration due to their small size [20]. However, clinical accomplishment of aptamers is trailing behind antibodies, with only a few FDA-approved aptamers available to date [21].

**Figure 1:**
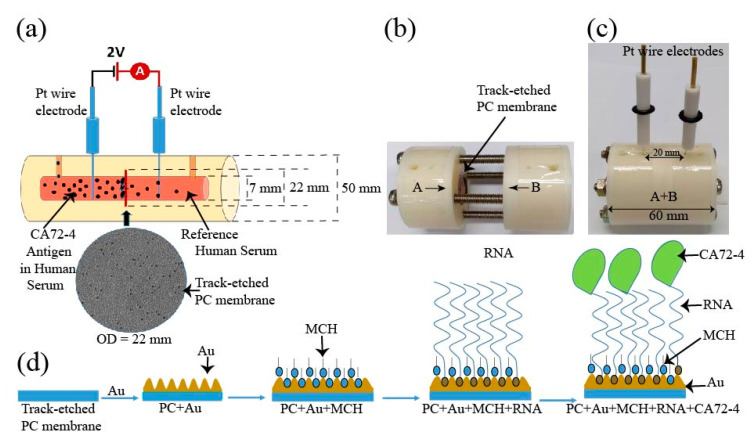
Schematic diagram of the design of the electrochemical sensor for detection of the CA72-4 cancer antigen. (**a**) Design of track-etched membrane-based electrochemical sensor. (**b**) The electrochemical cells before integration. (**c**) Integrated Teflon electrochemical cells with platinum (Pt) reference electrodes. (**d**) Surface modification of polycarbonate (PC) membranes and detection of cancer antigen CA72-4.

Herein, we developed a novel method to use RNA aptamers for early diagnosis of gastric cancer. The core component is a track-etched polycarbonate (PC) membrane conjugated with sequence-specific Oligo RNA aptamer for highly sensitive detection of cancer-marker antigen CA72-4. The membrane was built in a circuit, and then the electrical current passing through the membrane was measured in the presence of different concentrations of cancer antigen (Figure 1a–d). To explain the mechanism, we systematically studied the surface-enhanced Raman spectra, Fourier-transform infrared spectroscopy (FTIR), and X-ray photoelectron spectroscopy (XPS) of the membrane associated with Oligo RNA immobilization and different antigen concentrations. It was found that the targeted antigen was specifically bound to the aptamer and was trapped in the nanopores of the membrane, thereby influencing the electrical current of the circuit. In addition, the construction of the sensing device can be easily cleaned and reused. For more cost-effective sensing, it is convenient to replace Teflon housing with other commercially available materials such as glass and plastics. In measurement, we only used to change polycarbonate membranes for different measurement, which is not expensive. Our device is easy-to-use and portable, and, importantly, enables highly sensitive detection of cancer antigens with low detection limit, which may potentially pave a way for using aptamers for early diagnosis of cancer in clinic.

## 2. Materials and Methods

### 2.1. Materials

Nuclepore track-etched polycarbonate (PC) membranes were purchased from Whatman. Tris (2-carboxyethyl) phosphine hydrochloride (TCEP), 6-Mercapto-1hexanol (MCH), and human serum (from male AB clotted whole blood, USA origin, sterile-filtered) were ordered from Sigma Aldrich, Hong Kong. Phosphate-buffered saline solutions and RNase-free diethyl pyrocarbonate (DEPC)-treated water was from Thermo-Fisher Scientific HK. RNA Oligo with 5′ Thiol Modifier C6 S-S modification sequence/5ThioMC6-D/CGGGGGUGCGAAGGGGGGCAGAGGUUUGACGCGAGA-3 was obtained from Integrated DNA Technologies Pte. Ltd., Singapore, and CA72-4 antigen was purchased from ATCG Limited, Hong Kong.

### 2.2. Experimental Setup

The schematic of the construction of our cancer antigen sensor device is illustrated in Figure 1a. Two platinum (Pt) references electrodes were immersed into an electrochemical cell and connected to a source meter (Keithley 2420). In this work, the selectivity and sensitivity measurements were carried out at a constant voltage of 2 V. The inner diameter of the electrochemical cell holding the PC membrane was 7 mm and the distance between the two electrodes was 20 mm (Figure 1b). The electrochemical cell was constructed using a Teflon (PTFE-polytetrafluoroethylene) polymer tube. The PTFE Teflon tubes have exceptional chemical and corrosion resistance to stress-cracking and high stability at ambient temperature [22]. The electrochemical cell setup consisted of two cylindrical half cells (Figure 1b) used to grip a track-etched PC membrane as the sensing substrate and an O-ring was used to stop the solution from leaking from one cell to the other. The membranes had diameter, thickness, and pore size of 25 mm, 7 μm, and 50 nm, respectively. The overhaul distance of the electrochemical cell was 40 mm. The cells contained flow channels to replace the solution, and two cylindrical half-cells were tightened by screws as shown in Figure 1c. The membrane was coated with a thin layer of 10 nm gold by the evaporation method and then surface-modified with MCH to immobilize the Oligo RNA (Figure 1d). Subsequent changes in the electrical measurements with respect to time for different concentrations of CA72-4 were analyzed by Raman spectra, FTIR, and XPS spectroscopy, and the surface morphology and poles sizes were studied with scanning electron microscopy.

### 2.3. Oligo RNA Immobilization on Modified Gold-Coated PC Membrane

RNA Oligo with 5’ thiol modifier C6 S-S with a specific sequence was selected to capture CA72-4, the biomarker of gastric cancer cells. Although Oligo RNA is slightly more expensive than DNA sequencing, it can detect specific molecules and is more resistant to damage from UV light than DNA [23]. A standard purified RNA Oligo aptamer, 100 nmole and 36 bases pair, was used for antigen detection. Thiol-modified Oligos, which are protected by S-S bonds, need to be reduced in the protecting group before using. The TCEP (Tris [2-carboxyethyl] phosphine) treatment method was used for the reduction of the thiol-protected group. TCEP with an excess concentration of 30 mM was added to 300 µM Oligo and the mixture was allowed to settle for a couple of hours at room temperature for reducing the disulfide bond of thiol-modified Oligo RNA. The Oligo stock solution was stored at −20 °C. Before each use, the purity and concentration of Oligo RNA were double-checked using a Nanodrop spectrophotometer [24,25]. Prior to Oligo RNA immobilization, the PC membranes with a thin layer of gold coating were immersed in a 10 mM concentration of MCH for 1 h [26,27]. The membranes were then washed twice with the 1X-PBS buffer (pH 7.4) solution and distilled water. Immobilization of Oligo RNA onto the MCH-modified gold-deposited membrane was achieved by placing 10 µM of 50 µL of Oligo RNA on the membrane, followed by incubation at 37 °C for 60 min. The membrane was then washed with 1X PBS (pH 7.4) and distilled water to remove any free Oligo molecules [28,29] (Figure 1d). The shifts in currents and time measurement were studied to identify Oligo RNA attaching and targeted detection.

### 2.4. Electrical Response Measurements 

A stock 10 kU CA72-4 antigen solution was diluted with human serum to different concentrations including 4, 6, 7, 8, 10, 12, and 14 U/mL for sensitivity measurements [30]. The capability of a bio-receptor to detect a specific analyte in a sample containing other admixtures and impurities was checked by performing a selectivity test. The best example of selectivity is described by the interaction of an antigen with antibody, DNA, or RNA molecules [31] and hence CA19-9 is the best antigen for selectivity control examination. The reference range of CA 72-4 cancer antigen is 7 U/mL and the examination tests were carried out for different concentrations with a range from 4 to 14 U/mL [32].

### 2.5. Measurements and Data Acquisition

The track-etched nanopores were observed using a field-emission scanning electron microscope, QUANTA FEG-600, Philips. Surface-enhanced Raman spectra (SERS) measurements were performed using Renishaw in Via confocal Raman spectrometer with 633 nm laser. FTIR spectra were obtained using transmittance mode in a Perkin Elmer UATR Two spectrophotometer. XPS analysis was performed using PHI 5000 Versa Probe system with C1s peak of aliphatic carbon at 284.8 eV as the reference. The electrical measurements were carried out by using a Keithley 2612 Dual Source Meter under ambient conditions.

## 3. Results and Discussion

### 3.1. Setup of the Device

The surface modification process of the PC membrane for cancer antigen capturing is illustrated in Figure 1d. The membrane was first coated with a 10 nm-thick gold layer and sequentially modified with MCH, which is shown as blue particles appearing in the surface pores, which prevented the non-specific conjugation of RNA aptamers on the surface. The aptamer, which is shown as red particles, was to specifically capture cancer antigen CA72-4 and block the nanopores of the track-etched membrane that was made to decrease the rate of output current flow of the electric cell. The MCH layer prevented non-specific conjugation of the cancer antigen to the gold surface of the membrane. The pore size of the PC membrane was examined with SEM. Prior to gold coating, the pore size was approximately 50 nm (Figure S1a in Supplementary material), and after a 10 nm-thick gold coating, the average pore size reduced to approximately 30 nm (Figure S1b Supplementary material). 

**Figure 2:**
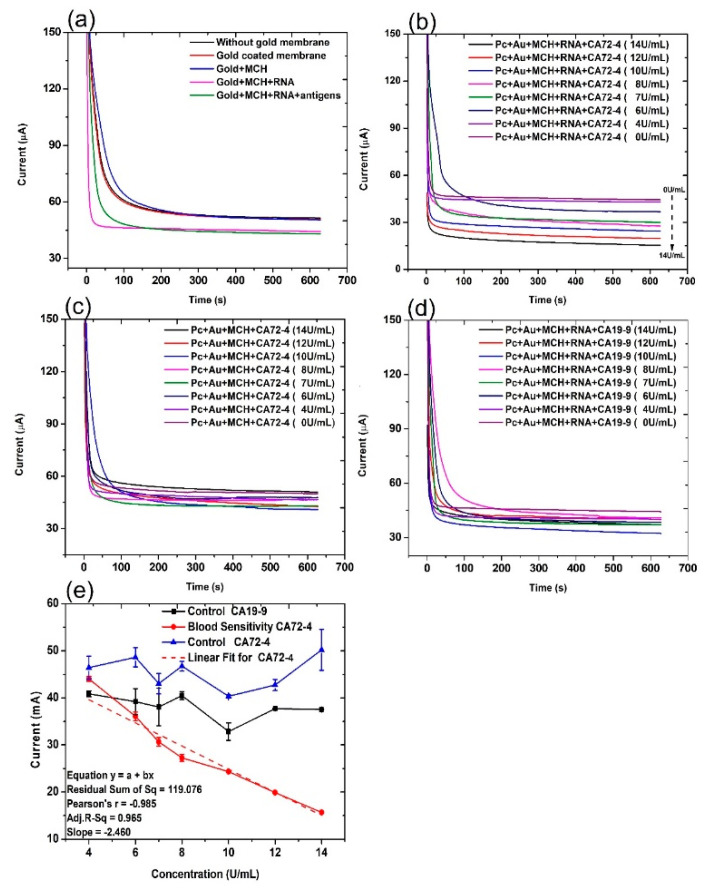
Current and time responses of the electrochemical sensor under different conditions. (**a**) Current and time responses of control samples and the sensor in the presence of 14 U/mL of CA 72-4 cancer antigen. (**b**) Current and time responses of electrochemical sensors in the presence of different concentrations of CA 72-4 cancer antigen. (**c**) Current and time responses of electrochemical sensor in the presence of different concentrations of CA 72-4 cancer antigen but without RNA aptamer. (**d**) Current and time responses of electrochemical sensors in the presence of different concentrations of CA 19-9 cancer antigen. (**e**) Calibration plots between the current of electrochemical sensor and the concentration of CA72-4 and CA19-9 in (**c**–**e**).

The current evolution with time of the electrochemical cell under different conditions was recorded under a constant voltage [33] of 2 V for 10 min (Figure 2). The control groups are the PC membrane without gold coating (PC), PC membrane with gold coating (PC + Au), PC membrane with gold coating and MCH modification (PC + Au + MCH), and PC membrane with gold coating and MCH and RNA modification (PC + Au + MCH + RNA). From Figure 2a, it can be seen that the current of all control groups is similar to or slightly higher than that of the circuit with PC membrane alone. In contrast, when we added cancer antigen CA72-4, the current values gradually decreased, with the concentration increasing from 0 to 14 U/mL (Figure 2b). The change of the current values is possibly due to the fact that the adsorption of CA72-4 to the RNA-modified PC membrane results in partial blockage of the nanopores, thereby reducing the ions flow in the channels with respect to the concentration of the added antigen. This cancer antigen detection is specific, which can be demonstrated by the relatively constant current upon addition of CA19-9 with a concentration from 0 to 14 U/mL (Figure 2d). The RNA coating is the key of the detection. If there is no RNA coating, the current is also relatively constant with the addition of CA 72-4 (Figure 2c).

### 3.2. Cancer Antigen Detection

For quantitative analysis, the relations between the measured current values and the concentrations of added antigen CA 72-4, and CA19-9 are plotted in Figure 2e. It is clear that the current gradually decreases with increasing CA 72-4 concentrations when the PC membrane is modified with CA 72-4 specific RNA aptamer. Clearly, the response curve indicates a linear relationship between the current value and antigen concentration. The linear equation used in this fitting is y = a + bx, residual sum of square = 119.076 with Pearson’s r value = −0.985. The linear regression R^2^ = 0.965 and the slope m = −2.460 µAU^−1^mL were calculated. The sensitivity of our sensor is 7.029 µAU^−1^mLcm^−2^ with the membrane-sensing surface area of 0.340 cm^2^ [34]. When we add another type of cancer antigen CA19-9 to the sensor, the current value does not significantly change with concentration variation (Figure 2e), indicating that the sensor has very good specificity in CA 72-4 detection. Electrical response studies for their magnified regions are exposed in Figure S2.

### 3.3. Mechanism of Cancer Detection

**Figure 3:**
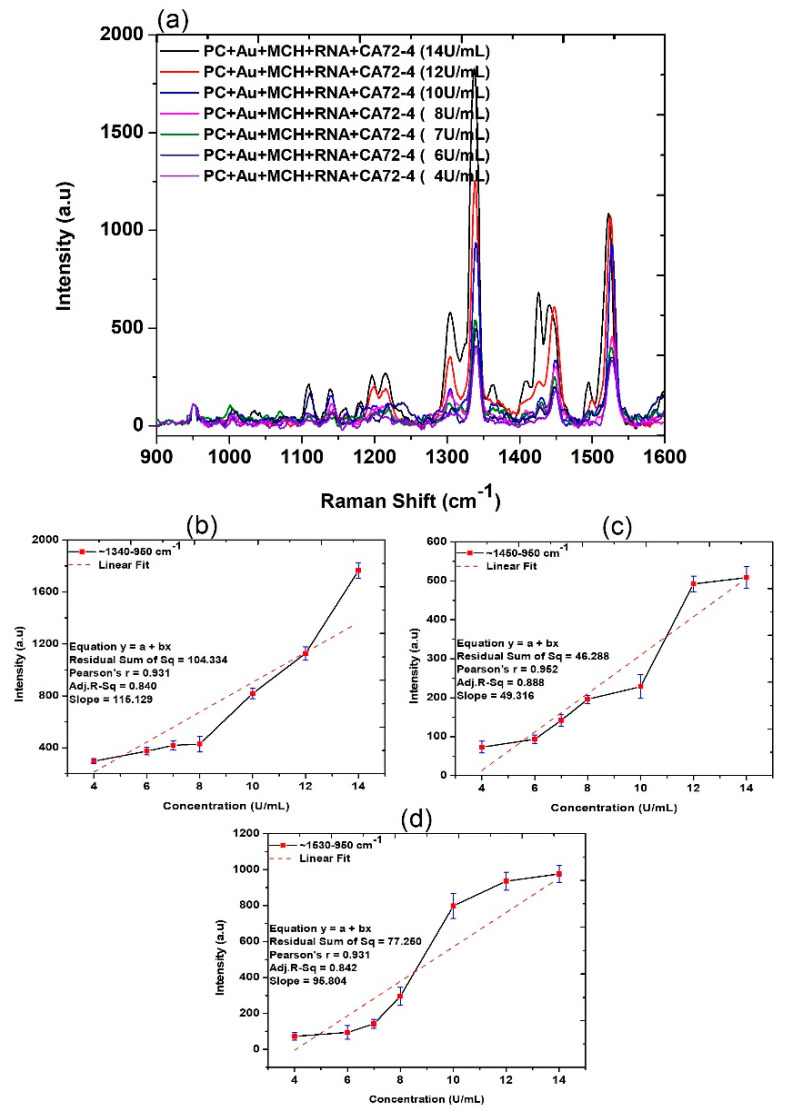
Raman spectra and quantitative analysis of adsorption of different concentrations of cancer antigen CA 72-4 on our electrochemical sensor. (**a**) Raman spectra of different concentrations of CA 72-4. (**b**) Plots of the intensity of the band calculated difference between two maxima regions of around 1340–950 cm^−1^. (**c**) Plots of the intensity of the band calculated difference between two maxima regions of around 1450–950 cm^−1^. (**d**) Plots of the intensity of the band calculated between two maxima regions of around 1530–950 cm^−1^.

After demonstrating the application of our device for highly sensitive measurement of cancer antigen CA72-4, we subsequently carried out systematic analysis to explore the mechanism of the detection. Firstly, the Raman scattering measurements of PC membranes under different conditions were obtained. The assignments of the Raman bands are listed in Table 1. The three strong peaks at 1530, 1450, and 1340 cm^−1^ are observed in Figure 3, and corresponding magnified regions are illustrated in Figure S3. The presence of antigen molecules with different concentrations can be observed at 1020–1032, 116–1178, 1219–1235, 1338–1345 and 1447–1460, 1530, and 1594–1608 cm^−1^ and RNA molecules were observed in 1309, 1326, and 1487 cm^−1^ in the same figure [35,36,37]. The quantitative studies of the Raman spectra (Figure 3b–d) were calculated from the difference between the intensities maximum and that of 950 cm^−1^, which indicated a reasonable linear relationship between the intensity increase and CA72-4 concentration at 1340, 1450, and 1530 cm^−1^, respectively. 

**Figure 4:**
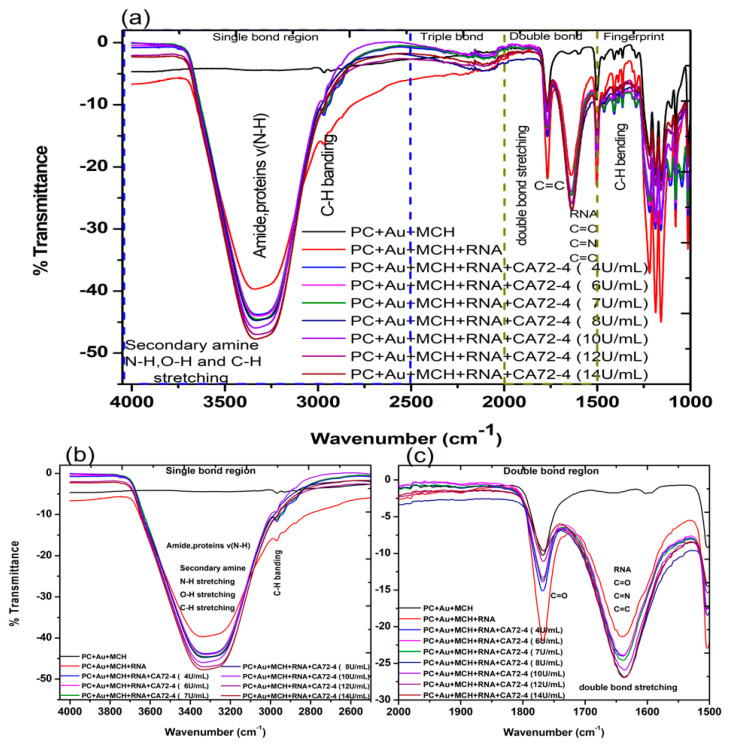
Fourier-transform infrared spectroscopy (FTIR) transmittance spectra of adsorption of different concentrations of cancer antigen CA 72-4 on our electrochemical sensor. (**a**) FTIR transmittance spectra for different concentrations (**b**) FTIR transmittance spectra for the single-bond region (**c**) FTIR transmittance spectra for the double-bond region.

We then investigated the FTIR transmittance spectra with the results presented in Figure 4a. All FTIR measurements were performed on hydrated samples to ensure the containing of biomolecules. The FTIR spectrum of the PC membrane with gold coating and MCH modification exhibited the following characteristic vibration bands: that of N-H, O-H, and C-H stretching, amide, and proteins v (N-H) was 3300 cm^−1^ (Figure 4b); stretching and vibration of C-H bonds of the CH_3_ group (sp^3^ C-H) was 2966 cm^−1^; (C=O) vibration for the carbonate group was 1760 cm^−1^; C=C vibration from the two phenol rings was 1504 cm^−1^ (Figure 4c); the 1250–1100 cm^−1^ range corresponded to the stretching and deformations of the O-C-O asymmetric carbonate group; 1080 cm^−1^ for the -CH_3_ vibrations; 1014 cm^−1^ was endorsed to an O-C-O symmetric carbonate group in stretching vibration mode; and the vibrational band for phenol rings [38] was attributed at 829 cm^−1^, as shown in Figure 4a [39]. 

The FTIR spectrum of the gold-coated membrane modified with MCH and Oligo RNA shows stretching vibrations from 3314 cm^−1^, corresponding to the stretching from the NH_2_ amines groups, (O-H) stretching from H-bonded alcohols, the phenols groups, and the C-H stretching vibration group (Figure 4a) [40]. The band at 1640 cm^−1^ (Figure 4b) of that spectrum is from amine groups present in the nitrogenous RNA bases. However, the bands at 1045 cm^−1^ are assigned to the vibration of C-C sugar ribose or associated with sulfite groups, which were found in phosphate groups of the RNA (-S=P). The band at 1227–1239 cm^−1^ are corresponding to the antisymmetric stretching vibration of phosphate groups (PO2-) [38,41,42].

**Figure 5:**
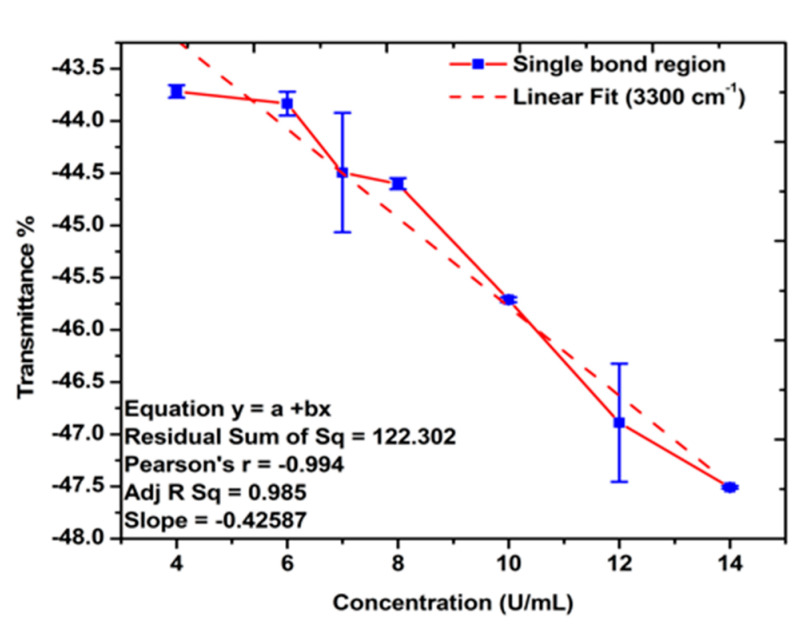
Linear relations between the transmittance % and concentration of antigen CA72-4. Quantitative analysis for the single-bond (C-H, C-N, N-H stretching, amide, proteins v (N-H), and C-H bending) region.

The FTIR spectra of the gold-coated membranes modified with MCH, Oligo RNA, and different concentrations of antigen CA72-4 show a decrease in transmittance values with increasing concentrations of antigen such as the secondary amine (proteins) groups N-H stretching bands at 3250–3400 cm^−1^ and N-H bending 1 amine (proteins) group at 1640 cm^−1^. The bands at 1020–1250 cm^−1^, 1250–1335 cm^−1^, and 1400–1500 cm^−1^ are the stretching vibration (C-N) of aliphatic amines (proteins), –N stretch aromatic amines (proteins) vibration groups, and C-C stretching aromatics (proteins) groups respectively [38,41,42,43]. Again, the quantitative studies of the peak intensities of the FTIR bands at 3300 cm^−1^ indicated (Figure 5) good linear relationships between FTIR intensities and the concentration of the antigens.

**Figure 6:**
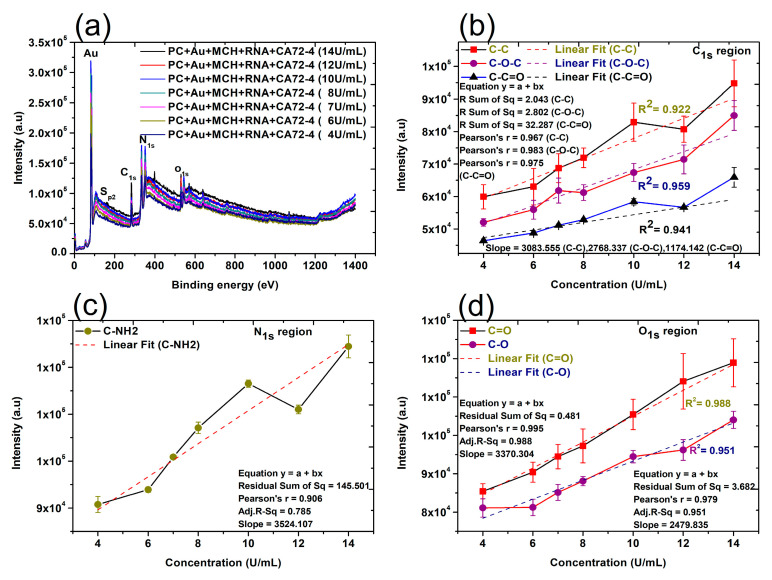
X-ray photoelectron spectroscopy (XPS) spectra and linear relation of adsorption of different concentrations of cancer antigen CA 72-4 on our electrochemical sensor. (**a**) The XPS spectra of adsorption of different concentrations of cancer antigen CA 72-4 on our electrochemical sensor. Plots of the concentrations of CA72-4 and the intensity of (**b**) the C-C, C-O-C, and C-C=O bonding C_1s_ region, (**c**) the C-NH_2_ bonding N_1s_ region, and (**d**) the C=O, C-O bonding O_1s_ region.

Finally, the changes of surface composition and chemical bonds on the surface of membranes were studied by XPS techniques. The XPS analysis provides valuable information about the composition of nanocomplexes and biomolecules [44]. In this work, XPS detected the shift of the peaks of the binding energy of different elements in the nanocomplexes after the addition of other components, such as gold nanoparticles, Oligo RNA, and antigens [45,46]. The quantitative analyses of the peak intensities of oxygen (O_1s_), nitrogen (N_1s_), and carbon (C_1s_) regions were performed (Figure 6). The quantitative analyses were also carried out for 284.8 eV (C-C), 286 eV (C-O-C), 288.5 eV (C-C=O), 531.5–532 eV (C=O), and 533 eV (C-O). Importantly, a reasonably good linear relation can always be found (Figure 6b–d), which is consistent with the results shown in the Raman and FTIR quantitative analyses. It is proved that the CA72-4 cancer antigens were binding with the specific aptamer sequence of the modified gold-coated polycarbonate membrane in a dose-dependent manner. Therefore, the concentration of the antigen can be conveniently determined with our technique. 

**Figure 7:**
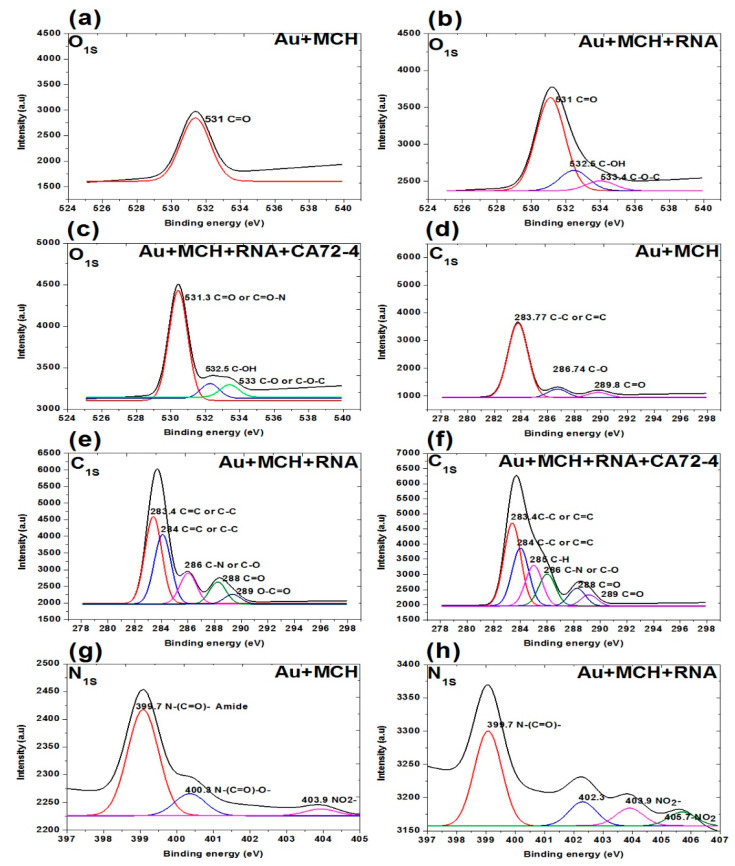
XPS analysis of adsorption of different concentrations of cancer antigen CA 72-4 on our electrochemical sensor. (**a**) O_1s_ analysis of MCH-modified gold-coated membrane. (**b**) O_1s_ studies on the Oligo-RNA membrane. (**c**) O_1s_ results of Oligo-RNA-modified and detected CA72-4. (**d**) C_1s_ region of MCH-modified gold membrane. (**e**) C_1s_ region of Oligo-RNA. (**f**) C_1s_ region of Oligo-RNA and CA72-4. (**g**) N_1s_ region of MCH-modified gold membrane. (**h**) N1s region of Oligo-RNA.

**Figure 8:**
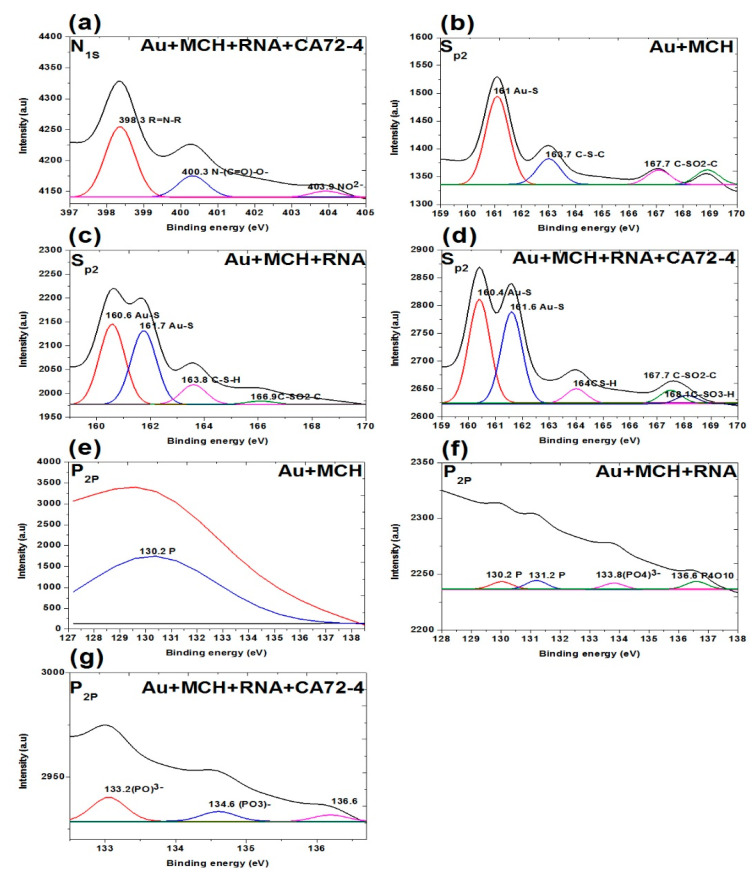
XPS analysis of adsorption of different concentrations of cancer antigen CA 72-4 on our electrochemical sensor. (**a**) N_1s_ analysis of Oligo-RNA-modified and detected CA72-4. (**b**) S_p2_ studies on MCH-modified gold membrane. (**c**) S_p2_ region of the Oligo-RNA membrane. (**d**) S_p2_ results of Oligo-RNA-modified and detected CA72-4. (**e**) P_2p_ region of MCH-modified gold membrane. (**f**) P_2p_ region of Oligo RNA. (**g**) P_2p_ region of Oligo RNA and CA72-4.

In addition, the XPS survey spectra of the sensing membrane are shown in Figure 7 and Figure 8. A strong Au peak, polycarbonate peak, C1s peak, and O1s peak; and weak P2p peak, S2p peak, and N1s peak were observed. The strong Au and polycarbonate peaks are the background of the membrane, and the oxygen peak can be attributed to some oxygen atoms on the membrane edges. Figure 7a–c shows the oxygen peaks of different membranes. The binding energies of 531 eV of the oxygen peaks of MCH-modified membrane (Figure 7a) are assigned to the C=O of the polycarbonate film. For the Oligo-RNA-modified membrane (Figure 7b), three oxygen peaks are observed at the binding energies of 533.4 eV, 532.5 eV, and 531 eV for the C-O-C peak, C-OH peak, and C=O peak, respectively [47]. Moreover, four oxygen peaks are observed in the XPS spectra of the Oligo RNA membrane with antigen CA72-4 (Figure 7c), where the binding energy of 531 for the C=O peak and other peaks are not defined.

Figure 7d–f shows the C1s spectra of the membranes. The three carbon peaks are 289.8 eV for C=O, 286.7 eV for C-O, and 283.7 eV for C-C functional groups for MCH-modified membranes. For the Oligo RNA-modified membrane, the five peaks at binding energies 283.4, 284.0, 286.0, 288.0, and 289.0 eV correspond to the C=C or C-C, C-N or C-O, C=O and C-C=O functional groups, respectively [48]. For the antigen CA72-4 and Oligo-RNA-modified membrane, the sixth peaks at the binding energies of 283.4, 284.0, 285.0, 286.0, 288.0 and 289.0 eV are corresponding to C-C, C=C, C-H, C-N, C-O, C=O, and O-C=O functional groups [48,49,50,51], which is in line with the FTIR results [52].

Figure 7g,h and Figure 8a show the N1s spectra of the membranes. The peaks at binding energies 399.4 eV, 400.3 eV, and 403.9 eV correspond to C=N-C, N-(C=O)-O, NO^2−^ for MCH-modified membrane. The peaks at 399.7 eV correspond to the N-(C=O)- functional group and the peaks at 403.9 and 405.7 eV are NO^2−^ functional group of the Oligo-modified membrane. For the targeted antigen CA72-4 and the Oligo-RNA-modified membrane, the three peaks are 389.3 eV for R=N-R, 400.3 eV for N-C=O, and 403.9 eV for the NO^2−^ functional groups [49,50,53].

Figure 8b–d shows the S2p spectra of the membranes. The three weak peaks are 161.0 eV for Au-S, 163.7 eV for C-S-C, and 167.7 eV for C-SO2-C functional groups. For the Oligo-RNA-attached membrane, the peaks at 160.0 eV and 161.7 eV are Au-S groups. The rest of the peaks include 163.8 eV for C-S-H and 166.9 eV for C-SO2-C functional group [54,55]. Here, we confirm the detachment of the thiol (-S-S-) bond encapsulating the Oligo RNA strand. Nevertheless, we cannot observe the thiol group (-S-S- bonds) in the sulphur region indicating the breakage of the thiol-group by TCEP and the attachment of Oligo RNA to the MCH-modified gold-coated polycarbonate (PC) membrane [54,55,56]. One additional peak at 168.1 eV was observed for the targeted antigen CA72-4 and Oligo-RNA-modified membrane.

Figure 8e–g shows the presence of phosphate group (P2p) on the membrane. A weak peak was observed at binding energy of 130.2 eV for the phosphorous group for the MCH-modified membrane [57]. The phosphorous functional group of the Oligo-RNA-modified membrane was observed at 130.2 eV, 131.2 eV, 133.8 eV, and 136.6 eV as shown in Figure 8f. For the targeted antigen CA72-4 and Oligo-RNA-modified membrane (Figure 8g), two more additional peaks appeared at the binding energies of 133.2 eV and 134.6 eV, confirming that Oligo RNA and the antigen were attached on the membrane [57,58].

As the results of quantitative studies, the performance of the sensing device is excellent in sensitivity and selectivity. However, further measurements are still required for distributing into the market. Compared with other techniques like paper-based biosensors, our technique is better in selectivity, sensitivity with low fabrication cost, simple, and portable in diagnostic stages [59,60]. In paper-based sensing technique, fabrication process is complex and stability is very low [61,62]. However, our technique only required gold coating on the track-etched membrane. All fabrication processes are simple and easy to perform except using the incubator system for aptamer immobilization.

## 4. Conclusions

In this study, we developed a PC membrane biosensor in conjunction with Oligo RNA aptamers for detection of a tumor marker, antigen CA72-4. This simple method exploits the direct immobilization of probe Oligo RNA molecules onto an MCH-modified gold-coated nanopore surface for capturing the targeted cancer biomarkers with both selectivity and sensitivity warranted. This simple approach does not require any complicated fabrication technique. Our device is able to detect antigen concentrations as low as 4 U/mL with a high sensitivity of 7.029 µAU^−1^mLcm^−2^. This sensing method for cancer-marker detection with track-etched membranes has not been previously reported to the best of our knowledge. The sensing mechanism relies on the RNA aptamer’s capture of the specific cancer antigen and resulting current change with the concentration of the antigen. Our device potentially stands as a powerful tool for quantitative detection of viruses, nanoparticles, proteins, and biomolecules.

## Figures and Tables

**Table 1:** The peak assignments observed in the Raman spectra.

Peaks	Assignments
1020–1032	Phenylalanine, CH in plane, CCassymmetric stretch (proteins)
1160–1178	Phenylalanine/tyrosine, CH bending, CO stretching, COH bending, Amide III (proteins)
1219–1235	Amide III, proteins.
1297	CH_2_ twist
1309	Adenine, RNA, Amide III (random coil, proteins)
1326	Guanine (RNA), CH deformation (proteins)
1338–1345	Adenine, Guanine (RNA), CH deformation (proteins)
1421–1430	Adenine, guanine, CH_2_ back bone (RNA)
1447–1460	CH_2_/CH_3_ bending, CH deformation (proteins)
1487	Adenine, Guanine, CH_2_ backbone (RNA)
1594–1608	Phenylalanine/Tyrosine C=C (proteins)
1621	Tyrosine/Tryptophan C=C (proteins)

## Data Availability

Not applicable.

## Supplementary Materials

The following are available online at https://www.mdpi.com/article/10.3390/s21082639/s1, Figure S1: Surface morphology study of PC membranes, Figure S2: Current and time response graph for magnified regions, Figure S3: Raman spectra for magnified regions.
