# Supplementary material for: Highly Sensitive and Cost-Effective Portable Sensor for Early Gastric Carcinoma Diagnosis
Source: Sensors (Basel). 2021 Apr 9;21(8):2639. doi: 10.3390/s21082639 (PMC8069728; doi:10.3390/s21082639)
Supplement: Supplementary file 1 [file sensors-21-02639-s001.pdf]

sensors

MDPI

Supplementary Materials

Highly Sensitive and Cost-Effective Portable Sensor for Early Gastric Carcinoma Diagnosis

Saw Lin Oo [1], Shishir Venkatesh [1], Vaithinathan Karthikeyan [1], Clement Manohar Arava [1], Spoorthy Pathikonda [2], Peter K. N. Yu [2], Terrence C.K Lau [3], Xianfeng Chen [4,*] and Vellaisamy A. L. Roy [5,*]

1 State Key Laboratory for THz and Millimeter Waves and Department of Material Science and Engineering, City University of Hong Kong, Kowloon, Hong Kong, China; kosawlinoo@gmail.com (S.L.O.); shishir.venkatesh@gmail.com (S.V.); kvecers@gmail.com (V.K.); clementmanohar7@gmail.com (C.M.A.)
2 Department of Physics, City University of Hong Kong, Kowloon, Hong Kong, China; spoorthy.709@gmail.com (S.P.); peter.yu@cityu.edu.hk (P.K.N.Y.)
3 Department of Biomedical Science, City University of Hong Kong, Kowloon, Hong Kong, China; chiklau@cityu.edu.hk
4 School of Engineering, Institute for Bioengineering, The University of Edinburgh, King's Buildings, Mayfield Road, Edinburgh EH9 3JL, UK
5 James Watt School of Engineering, University of Glasgow, Glasgow, G12 8QQ, UK
* Correspondence: Michael.Chen@ed.ac.uk (X.C.); Roy.Vellaisamy@glasgow.ac.uk (V.A.L.R.); Tel.: +44(0)131-650-2784 (X.C.); +44(0)141-330-0856 (V.A.L.R.)

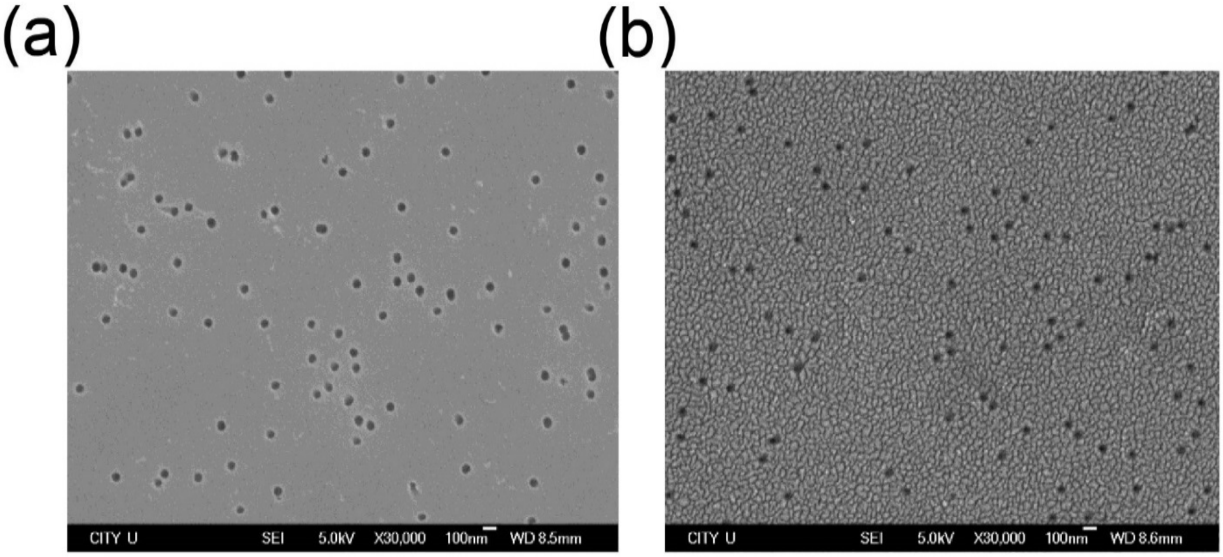

Figure S1. Surface morphology study of PC membrane with different surface modificatiion (**a**) PC membrane without gold coating (pore size ~50 nm) (**b**) PC membrane with gold coating (~30 nm).

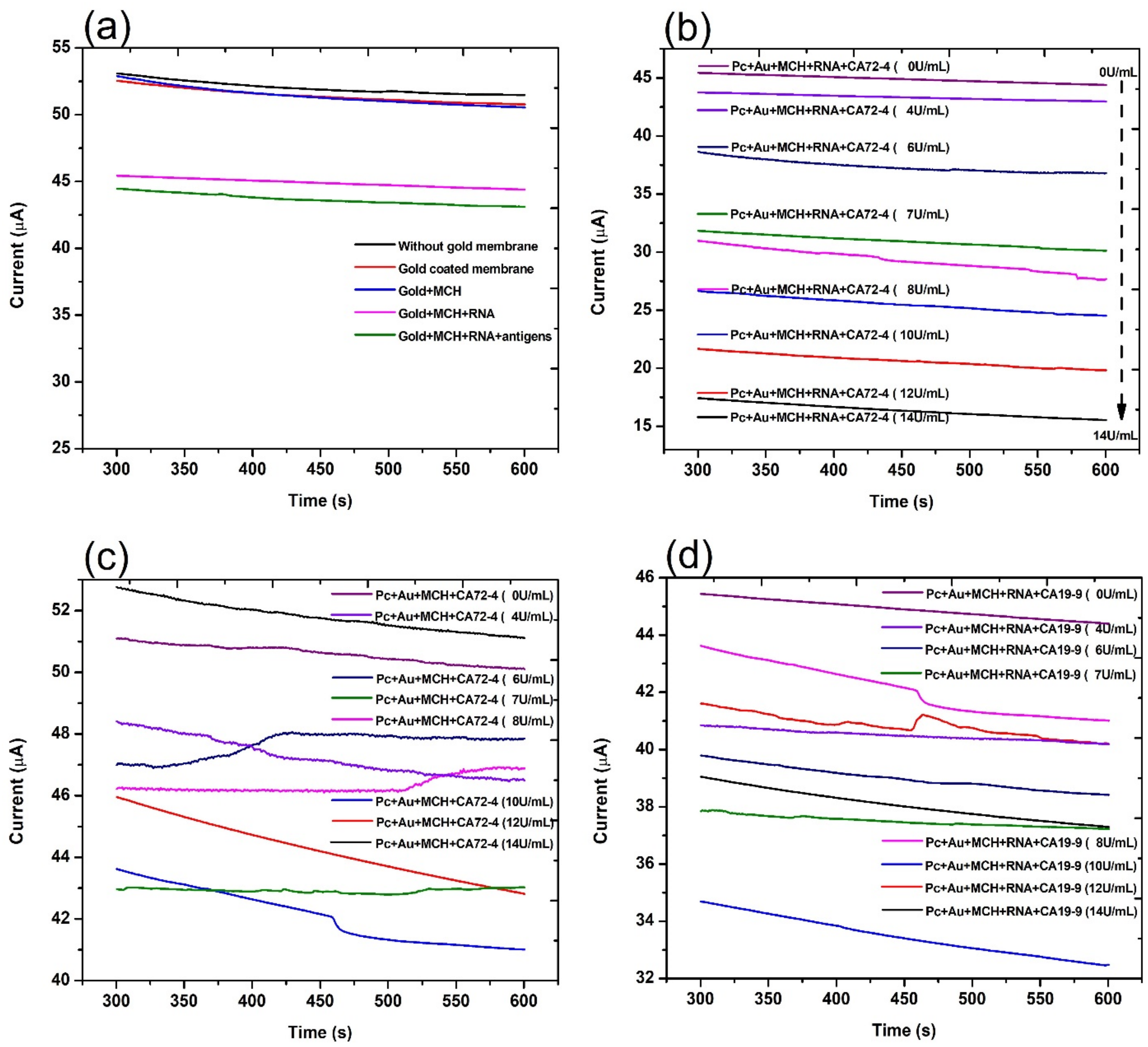

Figure S2. Current and time response graph for the mangified regions (**a**) Control background membrane. (**b**) Current and time response of sensing membrane. (**c**) Current and time electrical response of CA72-4 control membrane. (**d**) Current and time response of control CA19-9.

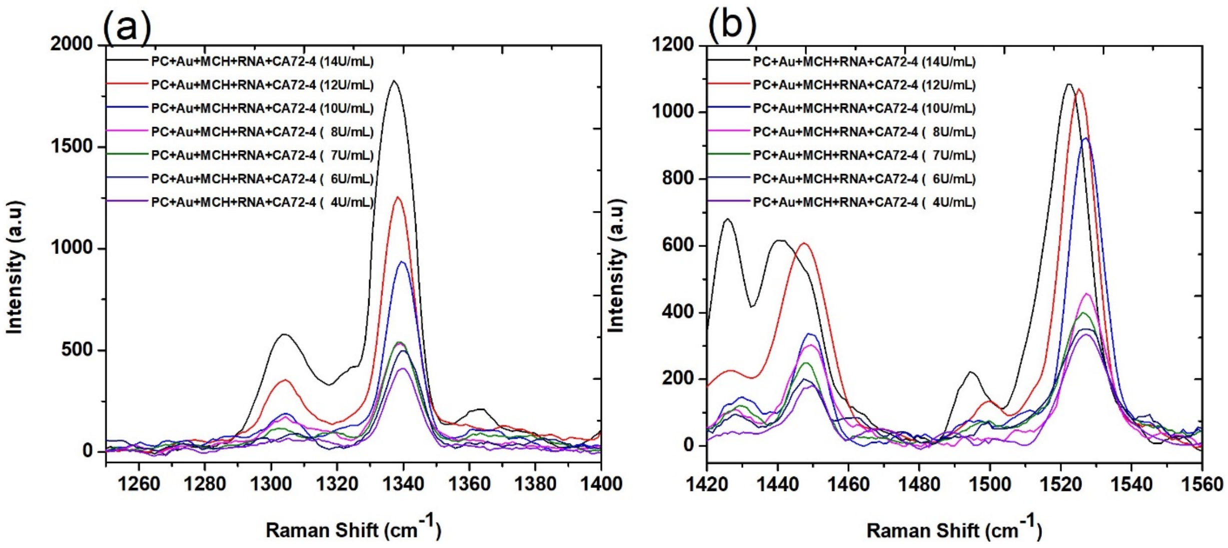

Figure S3. Raman spectra for magnified regions. (**a**) Raman shift spectra for 1250–1400 cm^{-1} region. (**b**) Raman shift spectra for 1420–1600 cm^{-1} region.
